# Reaction–Diffusion Model-Based Research on Formation Mechanism of Neuron Dendritic Spine Patterns

**DOI:** 10.3389/fnbot.2021.563682

**Published:** 2021-06-14

**Authors:** Yiqing Jia, Qili Zhao, Hongqiang Yin, Shan Guo, Mingzhu Sun, Zhuo Yang, Xin Zhao

**Affiliations:** ^1^Institute of Robotics and Automatic Information Systems, College of Artificial Intelligence, Nankai University, Tianjin, China; ^2^State Key Laboratory of Medicinal Chemical Biology, School of Medicine, Nankai University, Tianjin, China

**Keywords:** dendritic spine, Turing instability, reaction-diffusion model, branching morphogenesis, glioma

## Abstract

The pattern abnormalities of dendritic spine, tiny protrusions on neuron dendrites, have been found related to multiple nervous system diseases, such as Parkinson's disease and schizophrenia. The determination of the factors affecting spine patterns is of vital importance to explore the pathogenesis of these diseases, and further, search the treatment method for them. Although the study of dendritic spines is a hot topic in neuroscience in recent years, there is still a lack of systematic study on the formation mechanism of its pattern. This paper provided a reinterpretation of reaction-diffusion model to simulate the formation process of dendritic spine, and further, study the factors affecting spine patterns. First, all four classic shapes of spines, mushroom-type, stubby-type, thin-type, and branched-type were reproduced using the model. We found that the consumption rate of substrates by the cytoskeleton is a key factor to regulate spine shape. Moreover, we found that the density of spines can be regulated by the amount of an exogenous activator and inhibitor, which is in accordance with the anatomical results found in hippocampal CA1 in SD rats with glioma. Further, we analyzed the inner mechanism of the above model parameters regulating the dendritic spine pattern through Turing instability analysis and drew a conclusion that an exogenous inhibitor and activator changes Turing wavelength through which to regulate spine densities. Finally, we discussed the deep regulation mechanisms of several reported regulators of dendritic spine shape and densities based on our simulation results. Our work might evoke attention to the mathematic model-based pathogenesis research for neuron diseases which are related to the dendritic spine pattern abnormalities and spark inspiration in the treatment research for these diseases.

## Introduction

Dendritic spines are tiny protrusions on neuron dendrites which widely exist in the dendrites of higher animals and play an important role in the formation of most excitatory axodendritic synapses (Harris and Kater, 1994). The function of a spine is related to its shape (Kasai et al., 2003; Bourne and Harris, 2007). Traditionally, there are four basic shapes for dendritic spines: thin-type, stubby-type, mushroom-type, and branched-type (González-Tapia et al., 2016; Luczynski et al., 2016). Among them, thin dendritic spines show high plasticity and are related to learning, while mushroom dendritic spines show weak plasticity and are related to memory function. In addition, the density of spines directly influences the density of synapses. Researchers have found that pattern abnormalities of dendritic spine, especially the abnormal proportion of various types of dendritic spines and density variation of dendritic spines, were related to multiple nervous system diseases. For example, Pyronneau et al. reported an overabundance of thin-type spines, a kind of immature dendritic spines, in the somatosensory cortex of Fragile X syndrome model mice (Pyronneau et al., 2017). It has been reported that there are striatal dendrites with few dendritic spines in Parkinson's disease (McNeill et al., 1988). It was also been found that reduced dendritic spine density in individuals with schizophrenia (Glantz and Lewis, 2000; Sweet et al., 2008) and Huntington's disease (Richards et al., 2011). Also, it is recognized that dendritic spine loss is an early feature of Alzheimer's disease (Kommaddi et al., 2018; O'Neal et al., 2018). Thus, the exploration of shape and density factors of dendritic spines is of vital importance to understand the pathogenesis of these diseases, and further, search the treatment method for them.

The current research on dendritic spines pattern is mainly performed by statically observing the cerebral cortex in animals (Kommaddi et al., 2018; Ratliff et al., 2019). It has been confirmed that the pattern of dendritic spines is influenced by neuron activity (Portera-Cailliau et al., 2003; González-Tapia et al., 2016) and some substances, such as drebrin (Hayashi et al., 1996), Rho GTPase Rac1 (Pyronneau et al., 2017) and F-actin (Kommaddi et al., 2018). The above researches usually only proposed one factor of dendritic spine patterns once while the pattern formation of dendritic spines is a dynamic process involving a variety of chemical reactions that are regulated by multiple factors. In summary, there is still a lack of systematic study on the mechanism of pattern formation showing influences of multiple factors on the formed pattern of dendritic spines.

Mathematic modeling on dendritic spines development has become an important tool to study the structure and plasticity of dendritic spines in recent years. For example, Kasai et al. used the volume of dendritic spines as an index to measure the structure of dendritic spines and applied the Brownian motion model to simulate the volume of dendritic spines, exploring the close relationship between spine structure and function (Kasai et al., 2010). The Brownian motion model describes a random phenomenon, but the pattern formation of dendritic spines is a process regulated by gene and environment instead of a random process, making that model unsuitable for simulating the pattern formation. Besides, Miermans et al. simulated dendritic spine membranes during shape alternation using the Canham-Helfrich energy functional, which is used to describe the relationship between the bending rigidity of the membrane and the force generated by the cytoskeleton (Miermans et al., 2017). Their results demonstrate that the cytoskeleton is a key factor in determining the shape of dendritic spines, but this model lacks an explanation for the change in cytoskeletons, and their hypothesis about the approximate rotational symmetry of dendritic spines seems inapplicable to branched-type dendritic spines. Varner et al. explained the process of epithelial cell formation patterns using four mechanisms: cell division, cell insertion, cell deformation, and media filling (Varner and Nelson, 2014). However, these explanations cannot be applied in the study of sub-cellular structures such as dendritic spines.

In Turing theory, if the chemical substances involved in the interaction have diffusion, the original equilibrium state will be broken, which is called Turing instability (Turing, 1952). The reaction-diffusion model (Gierer and Meinhardt, 1972; Meinhardt, 1976), based on Turing's theory, illuminates the reactions between chemical substances in developing biological systems. It has been utilized to simulate Pomacanthus skin stripe patterns (Kondo and Asai, 1995), vascular mesenchymal cells patterns (Garfinkel et al., 2004), mouse limb development (Miura et al., 2006), lung branching patterns (Guo et al., 2014a; Hagiwara et al., 2015), and self-organizing morphogenesis (Okuda et al., 2018; Landge et al., 2020). In our previous work, side branching and tip branching of the lung were investigated using the reaction-diffusion model, which was verified by spatiotemporal parameters (Guo et al., 2014a). However, the patterns developed in previous work were not enough to describe the complex patterns in dendritic spines. Because different from the obtained side branches which were equally spaced, the dendritic spines studied in this paper are usually uneven. In spite of its potential use in simulate branching patterns, the strong non-linearity of the reaction-diffusion model makes it difficult to intuitively draw the relationship between parameter values and simulation results, which is inconvenient for the analysis of the inner mechanism of the model. Addressing this problem, dispersion relation was used to analyze Turing instability (Guo et al., 2014b; Saleem and Ali, 2018) to prove the mathematical mechanism of the simulation results. In previous research, we have investigated the mathematic mechanism through Turing instability analysis and found that different Turing wavelengths are underlying the different patterns in a lung (Xu et al., 2017). However, the relationship between Turing wavelength and branch density has not been investigated yet.

This paper reinterpreted the traditional reaction-diffusion model through the introduction of exogenous activator term and exogenous inhibitor term to simulate the formation process of dendritic spine, and further, study the factors affecting spine patterns. All four spine shapes, mushroom-type, stubby-type, thin-type, and branched-type, were reproduced using the model. Further, we found that the consumption rate of substrates by the cytoskeleton regulates the shape. Secondly, we found that the addition of an exogenous activator causes the spines to become denser, while the addition of an exogenous inhibitor causes the spines to become sparser, which provided a potential explanation for the anatomical results that spine decrease in hippocampal CA1 in SD rats with glioma. Finally, through Turing instability analysis, we found that Turing wavelength variation is the deep mathematical mechanism behind above parameters regulating spine density. Namely, the addition of an exogenous activator decreases the Turing wavelength, causing the density of the dendritic spines to increase, while the addition of an exogenous inhibitor increases the Turing wavelength, causing the density of the dendritic spines to decrease. Finally, the deep regulation mechanisms of several regulators of dendritic spine shape and density reported in other references were discussed based on our simulation results. We hope that our work could evoke attention to the mathematic model-based research for neuron diseases related to the dendritic spine pattern abnormalities and spark inspiration in the treatment research for these diseases.

## Materials and Methods

### Reaction-Diffusion Model

The reaction-diffusion model is defined by Equation (1) (Meinhardt, 1976). It is a group of partial differential equations describing the reactions between activator *A*, inhibitor *H*, substrate *S*, and cytoskeleton *Y*.

(1) $$\begin{array}{l} \{\begin{array}{c} \frac{\partial A}{\partial t}=\frac{cA^{2}S}{H}-\mu A+\rho_{A}Y+D_{A}\nabla^{2}A\\ \frac{\partial H}{\partial t}=cA^{2}S-\upsilon H+\rho_{H}Y+D_{H}\nabla^{2}H\\ \frac{\partial S}{\partial t}=c_{0}-\gamma S-\varepsilon YS+D_{S}\nabla^{2}S\\ \frac{\partial Y}{\partial t}=dA-eY+\frac{Y^{2}}{1+fY^{2}} \end{array}\;. \end{array}$$

**Figure 1:**
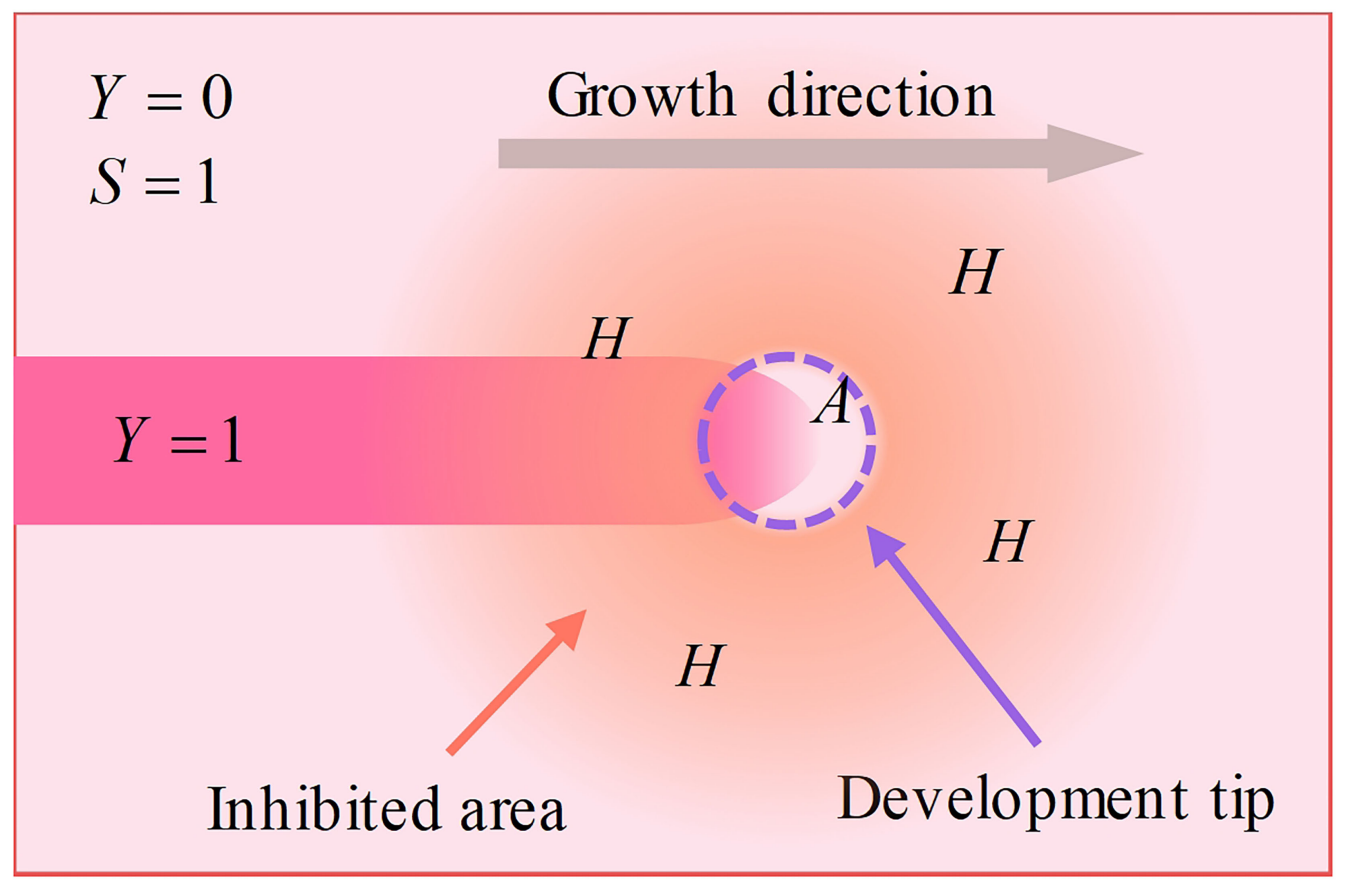
Schematic of the development process. The neuron expresses activators and inhibitors. Activators gather at the tip, while inhibitors diffuse into the surrounding area due to a higher diffusion rate, making only the tip develops. This mechanism makes dendritic spines grow in a certain direction instead of exhibiting isotropous growth.

The reaction-diffusion model illuminates the reactions between chemical substances in developing biological systems. According to this model, neurons express activators (at a rate ρ_A_) and inhibitors (at a rate ρ_H_). Activators behave with self-catalysis (at a rate *c*) and catalyze inhibitors (at a rate *c*), while inhibitors inhibit activators. Simultaneously, activators and inhibitors behave with degradation and diffusion (activators degrade at a rate μ and diffuse at a rate *D*_A_, whereas inhibitors degrade at a rate υ and diffuse at a rate *D*_H_). High concentrations of activator accelerate the polymerization of cytoskeletons, inducing the development of dendritic spines. Because the diffusion rate of inhibitors is higher than that of activator, the polymerization of the cytoskeleton in the growth center is accelerated, and the polymerization of the cytoskeleton outside the growing center is inhibited. Thus, the dendritic spine grows in a certain direction, instead of displaying isotropous growth. The neuron creates substrate (at a rate *c*_0_), while the cytoskeleton consumes substrate (at a rate ε). Substrate accelerates the catalysis of the activator. Similarly, the substrate behaves via degradation and diffusion (degrades at a rate γ and diffuses at a rate *D*_S_), as well. Because the synthesis of the cytoskeleton consumes substrate, the peak concentration areas of activators and inhibitors, as well as the cytoskeleton, move in the direction of high substrate concentrations (Figure 1).

The development patterns of dendritic spines are determined by the neuron activity (Bloodgood and Sabatini, 2005) and the exogenous substances. The neuron activity is described by the rate of substrate consumed by the cytoskeleton (ε) in our model. Exogenous substances include exogenous activators and exogenous inhibitors (Kommaddi et al., 2018). To describe the influence of exogenous substances, we added the exogenous activator term (δ_A_) and the exogenous inhibitor term (δ_H_) into the reaction-diffusion model:

(2) $$\begin{array}{l} \{\begin{array}{c} \begin{array}{ccc} \frac{\partial A}{\partial t} & = & \frac{cA^{2}S}{H}-\mu A+\left(\rho_{A}+\delta_{A}\right)Y+D_{A}\nabla^{2}A\\ \frac{\partial H}{\partial t} & = & cA^{2}S-\upsilon H+\left(\rho_{H}+\delta_{H}\right)Y+D_{H}\nabla^{2}H \end{array}\\ \begin{array}{ccc} \frac{\partial S}{\partial t} & = & c_{0}-\gamma S-\varepsilon YS+D_{S}\nabla^{2}S\\ \frac{\partial Y}{\partial t} & = & dA-eY+\frac{Y^{2}}{1+fY^{2}} \end{array} \end{array}\;. \end{array}$$

The new model includes 16 parameters, most of which are fixed parameters, such as reaction-term parameter *c*, degradation-term parameters μ, υ, and γ, diffusion-term parameters *D*_A_, *D*_H_, and *D*_S_, and growth-term parameters *d, e*, and *f* . The values of fixed parameters are decided by the chemical characteristics of substances or cells, and the model has been proven to be robust to perturbations of fixed parameters (Murray, 1982). The other parameters are variable (ρ_A_, δ_A_, ρ_H_, δ_H_, *c*_0_, and ε), whose values depend on the condition of the development system. In this work, we studied the effect of the neuron activity and the exogenous substances on dendritic spines. Thus, we set parameters δ_A_, δ_H_, and ε in Equation (2) as variable parameters.

The values of parameters were set according to previous research in lung branching patterns. In previous work, we set the values of parameters as: *c* = 0.002, μ = 0.18, υ = 0.04, ρ_A_ = 0.063, ρ_H_ = 0.00005, *c*_0_ = 0.02, γ = 0.02, ε = 0.045, *D*_A_ = 0.02, *D*_H_ = 0.32, *D*_S_ = 0.06, *d* = 0.0033, *e* = 0.1, and *f* = 10. We verified the consistency of the mathematical model under certain parameters with the actual biological process by converting the time and space in the numerical simulation and comparing them with the spatiotemporal scale of real lung development (Guo et al., 2014a). The values of fixed parameters and the value ranges of variable parameters in the lung branching model provide references in our new model.

### Numerical Simulation

**Figure 2:**
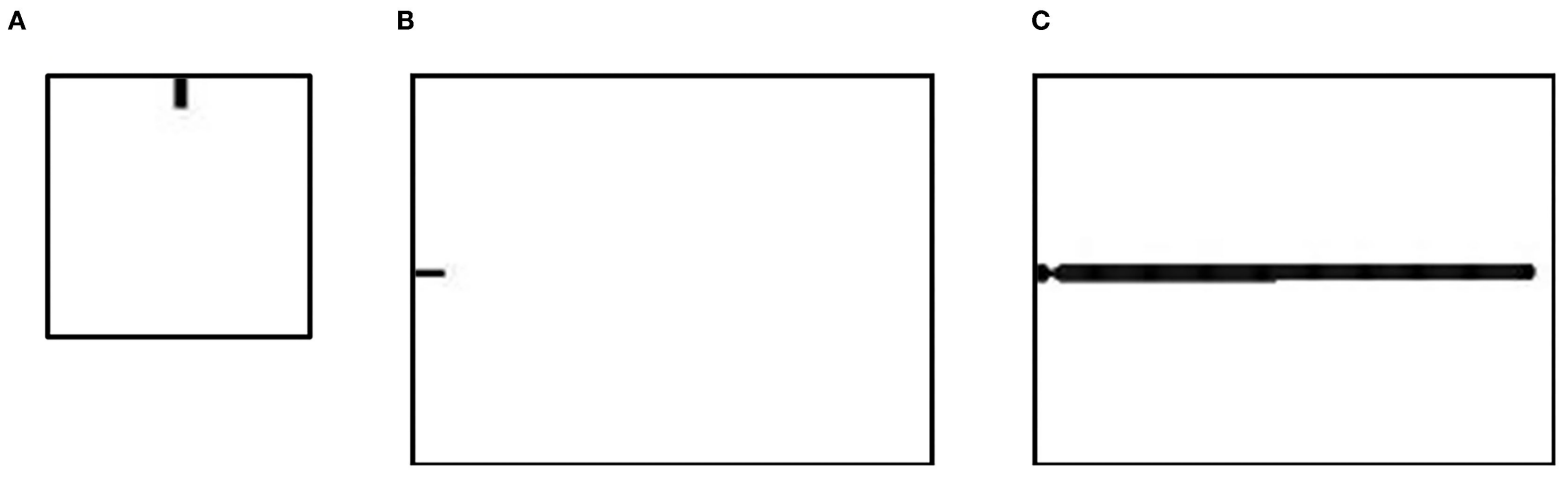
The original state of the spine simulation and the dendrite simulation. **(A)** The original state of the spine simulation is used to simulate a single spine in different conditions. Simulations were performed on a 100× 100 grid. The grid size of space is 0.3. Fixed parameters in Equation (2): *c* = 0.002, μ = 0.16, υ = 0.04, ρ_A_ = 0.01, ρ_H_ = 0.00005, *c*_0_ = 0.02, γ = 0.02, *D*_A_ = 0.02, *D*_H_ = 0.26, *D*_S_ = 0.06, *d* = 0.0035, *e* = 0.1, and *f* = 10. **(B)** The first step in the dendrite simulation is used to simulate the dendrite trunk. Simulations were performed on a 150× 200 grid. The grid size of the space is 0.3. Parameters in Equation (2): *c* = 0.002, μ = 0.16, υ = 0.04, ρ_A_ = 0.03, ρ_H_ = 0.0001, δ_A_ = 0, δ_H_ = 0, *c*_0_ = 0.02, ε = 0.017, γ = 0.02, *D*_A_ = 0.02, *D*_H_ = 0.26, *D*_S_ = 0.06, *d* = 0.0035, *e* = 0.1, and *f* = 10. **(C)** The second step in the dendrite simulation grows from **(A)** and is used to simulate spines in different conditions. Fixed parameters in Equation (2): *c* = 0.002, μ = 0.16, υ = 0.04, ρ_A_ = 0.02, ρ_H_ = 0.00005, *c*_0_ = 0.05, γ = 0.02, *D*_A_ = 0.02, *D*_H_ = 0.26, *D*_S_ = 0.06, *d* = 0.0035, *e* = 0.1, and *f* = 10. In **(A,B)**, black regions (*A* = 2, *H* = 0.02, *S* = 1, *Y* = 1) represent a part of a neuron, and white regions (*A* = 0.001, *H* = 0.001, *S* = 1, *Y* = 0) represent the environment surrounding the neuron.

In this work, we investigated the factors of shape and density of spines using a reaction-diffusion model on different spatial scales. First, we simulated a spine to explore the influence of model parameters on the shape of the spine (Figure 2A). This simulation was performed on a 100 × 100 grid, and the original state was a 10 × 5 pixels rectangular area. Second, we simulated a dendrite with spines to explore the influence of model parameters on the density of spines (Figures 2B,C). This simulation was performed on a 150 × 200 grid, and the original state was a 5 × 10 pixels rectangular area (Figure 2B). Then, a dendrite developed under certain conditions (Figure 2C).

### Turing Instability Analysis Method

To verify the simulation results with mathematics, we explored Turing patterns underlying dendritic spine patterns with our previously developed decoupling method (Guo et al., 2014b). The substrate and cytoskeleton are considered dependent variables of time and space, written as *S*(x, y, t) and *Y*(x, y, t). Then, we put these variables into Equation (2) as parameters and obtained the model of an activator-inhibitor system as:

(3) $$\begin{array}{l} \{\begin{array}{c} \frac{\partial A}{\partial t}=\frac{cA^{2}S\left(x,y,t\right)}{H}-\mu A+\left(\rho_{A}+\delta_{A}\right)Y\left(x,y,t\right)+D_{A}\nabla^{2}A\\ \frac{\partial H}{\partial t}=cA^{2}S\left(x,y,t\right)-\upsilon H+\left(\rho_{H}+\delta_{H}\right)Y\left(x,y,t\right)+D_{H}\nabla^{2}H \end{array}\;. \end{array}$$

Branching is a system that can grow and form stable mode, which corresponds to the damped oscillation system of mathematical model. Some points in S-Y space correspond to damped oscillatory systems. The set of these points is called Turing instability space, and the wavelength of damped oscillation system is called Turing wavelength (Turing, 1952). According to its definition, the mathematical expression of Turing space can be calculated. The detailed derivation process is in our previous work (Xu et al., 2017).

To explore dendritic spine development patterns according to Turing instability, a scheme was performed according to the following steps.

- Choose an interesting point (the branching point in the branched spine or a random point on the central axis in others) in a simulation result and plot the *S*-*Y* curve of this point.
- Calculate the Turing instability space using Equation (3).
- Find the intersection of the *S*-*Y* curve and Turing instability space.
- According to the form of the solution of Equation (2), we have

(4) $$\begin{array}{l} \text{w}\left(\text{r},\text{t}\right)\sim \underset{k}{\sum }c_{k}e^{\lambda_{k}t}W_{k}\left(r\right), \end{array}$$

and calculate the dispersion relation:

(5) $$\begin{array}{l} \lambda =\lambda \left(k\right). \end{array}$$

- Record the maximum of the real part of the eigenvalue (λ_m_) and corresponding wavenumbers (*k*_m_).
- Calculate Turing wavelength (Λ) of the point in Step 1:

(6) $$\begin{array}{l} \Lambda =\frac{2\pi }{k_{m}}. \end{array}$$

We used Turing instability analysis to explore the difference of mathematical mechanism behind different patterns of dendritic spines in section Turing Instability Underlying Dendritic Spines.

### Anatomy of Hippocampal CA1 in SD Rat

In this study, images from Golgi-Cox-stained brain slices from SD rats were compared with simulation results. Golgi-Cox staining was carried out with a commercial Golgi staining kit (Keyijiaxin, Tianjin, China). As soon as they were taken from the skulls, the brains were stored in Golgi-Cox staining solution in a dark place for 2 weeks, and the solution was replaced at intervals of 48 h. Then, brain slices were produced using a vibratome (VT 1000S, Leica, Germany) with a thickness of 150 μm. The slices were placed on slides covered with 2% gelatine. Next, the slices were dyed with ammonia for 60 min; washed with water three times; fixed with Kodak film for 30 min; and then washed with water, dehydrated, cleared, and mounted. Later, dendritic spines in the CA1 region of the hippocampus were imaged under the 100 × objective lens with a digital camera. Dendritic trees were detected along CA1 tertiary dendrites derived from secondary dendrites, which started at their point on the primary dendrite. All animal experiments were approved by the Animal Research Ethics Committee, School of Medicine, Nankai University and were performed in accordance with the Animal Management Rules of the Ministry of Health of the People's Republic of China.

## Results

### Dendritic Spine Shape Factors Research Based on Reaction-Diffusion Model

There are four traditional types of dendritic spines: mushroom-type, stubby-type, thin-type, and branched-type (González-Tapia et al., 2016; Luczynski et al., 2016). In order to research the factors of dendritic spine shape, we firstly proposed a classification method of spine shape based on real spine microimages. Then, we classified a spine simulated by our reaction-diffusion model and found the change rule of dendritic spine shape in different conditions.

#### Classification Method of Dendritic Spine Shape

**Figure 3:**
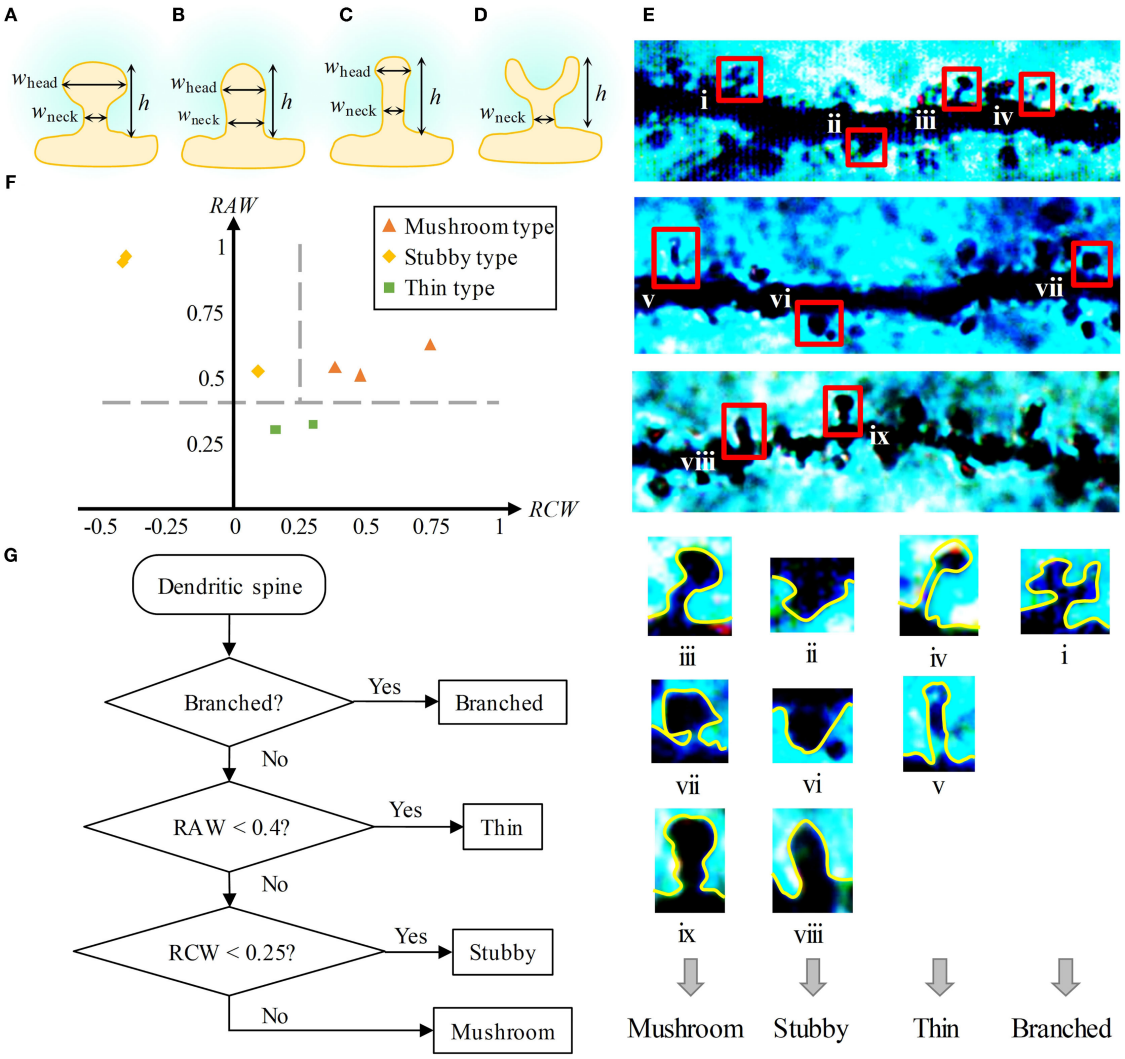
Metrics of dendritic spine shape. **(A–D)** Three geometric qualities of dendritic spines, namely, the height (*h*), the extreme width of the head (*w*_head_), and the extreme width of the neck (*w*_neck_). **(A)** is a mushroom-type spine, **(B)** is a stubby-type spine, **(C)** is a thin-type spine, and **(D)** is a branched-type spine. For a branched-type spine, the extreme width of the head is meaningless. **(E)** Top: Golgi-Cox staining of brain slices from SD rat hippocampal CA1. Four types of spines emerge in the images. Bottom: We found nine dendritic spines (including three mushroom-type spines, three stubby-type spines, two thin-type spines, and one branched-type spine) in the above images. **(F)** Two-metrics distribution of mushroom-type, stubby-type, and thin-type spines. Three types can be classified by the criteria RAW = 0.4 and RCW = 0.25. **(G)** Flow chart of the classification method of dendritic spine shape.

At present, the classification methods of dendritic spines shape are qualitative, expert experience-required. To study the shape of dendritic spines quantitatively, metrics to classify dendritic spines need to be determined. Given a branched-type dendritic spine is easy to identify, here we only propose a classification method for the three types of non-branched spines. First, we measured three geometric qualities of a dendritic spine, namely, the height (*h*), the extreme width of the head (*w*_head_), and the extreme width of the neck (*w*_neck_), as shown in Figures 3A–D. Then, based on these three values, we constructed two following dimensionless metrics:

- Relative average width (RAW) measures the overall thickness of spines, defined as

(7) $$\begin{array}{l} \text{RAW}=\frac{\left(w_{head}+w_{neck}\right)}{2h}. \end{array}$$

- Relative constriction width (RCW) measures the difference between the head width and the neck width, defined as

(8) $$\begin{array}{l} \text{RCW}=\frac{\left(w_{head}-w_{neck}\right)}{h}. \end{array}$$

We calculated the RAWs and RCWs of eight dendritic spines (including three mushroom-type spines, three stubby-type spines, and two thin-type spines, shown in Figure 3E). Thin-type spines have a thin head and neck, so the value of RAW is small. Both the head and neck of stubby-type spines are thick, and the head is thinner or slightly thicker than the neck, so for them, the value of RAW is usually large, and the value of RCW is small or even negative. For the mushroom-type spines, usually have a large head and a thin neck, their values of RAW and RCW are both large. Based on the above analysis, we set the metrics for three types of dendritic spines. As shown in Figure 3F, the shape differences among the three types of spines are obvious. We chose RAW = 0.4 and RCW = 0.25 as two criteria to classify the three types.

Finally, we presented a flow chart to distinguish the shapes of dendritic spines (Figure 3G). First, if the dendritic spine has a branching structure, it is recognized as a branched-type spine. Second, if the RAW value is lower than 0.4, it is regarded as a thin-type spine. Finally, if the RCW value is lower than 0.25, it is identified as a stubby-type spine. Otherwise, it is recognized as a mushroom-type spine.

#### Consumption Rate of Substrate Dominates the Spine Shape Based on the Reaction-Diffusion Model

In our previous simulation, the rate that substrates are consumed by cells has been shown to play an important role in the branching pattern (Xu et al., 2017). Thus, we assumed that the consumption rate of substrates, namely, the neuron activity, has an effect on the spine shape. To verify this assumption, we performed the following single-spine simulations.

**Figure 4:**
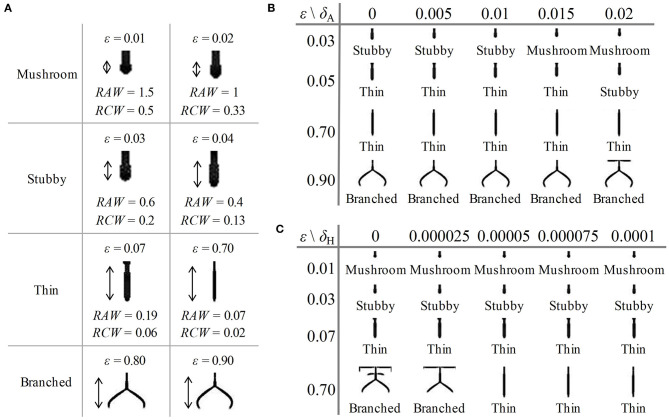
Influence of parameters on the shape of dendritic spines. **(A)** Some of the results of spine simulations for the condition of δ_A_ = 0.01, δ_H_ = 0.00005, 0.01 ≤ ε ≤ 0.9 (with different amplification factors). Arrows mark the newborn parts of spines growing from the original state in Figure 2C. With the enhancement of neuron activity, the values of RAW and RCW decrease, and all four shapes are obtained. The shape can be mushroom (0 < ε ≤ 0.02), stubby (0.02 < ε ≤ 0.04), thin (0.04 < ε ≤ 0.7) or branched (0.7 < ε ≤ 0.9) types. **(B)** The results of spine simulation in the condition of δ_A_ = 0, 0.005, 0.01, 0.015, and 0.02, δ_H_ = 0.00005, ε = 0.03, 0.05, 0.7, and 0.9. With the addition of an exogenous activator, the stubby-type spine becomes mushroom-type, and the thin-type spine becomes stubby-type. **(C)** The results of spine simulation in the condition of δ_A_ = 0.01, δ_H_ = 0, 0.000025, 0.00005, 0.000075, and 0.0001, ε = 0.01, 0.03, 0.07, and 0.7. With the addition of an exogenous inhibitors, the branched-type spine becomes thin-type. In **(A–C)**, fixed parameters in Equation (2): *c* = 0.002, μ = 0.16, υ = 0.04, ρ_A_ = 0.01, ρ_H_ = 0.00005, *c*_0_ = 0.02, γ = 0.02, *D*_A_ = 0.02, *D*_H_ = 0.26, *D*_S_ = 0.06, *d* = 0.0035, *e* = 0.1, and *f* = 10.

First, to investigate the influence of the consumption rate of substrates (ε) on the shape of dendritic spine, we adjusted the value of parameter ε in Equation (2). We varied the value of ε from 0.01 to 0.9, and part of the obtained results are shown in Figure 4A (with different amplification factors) (also see Supplementary Videos 1–4, respectively). As the value of ε increases, both RAW and RCW values decrease, and the dendritic spine shapes sequentially undergoes mushroom (0 < ε ≤ 0.02), stubby (0.02 < ε ≤ 0.04), thin (0.04 < ε ≤ 0.7), and branched (0.7 < ε ≤ 0.9) forms. All four dendritic spine shapes can be obtained with an increase in the consumption rate of substrates. This result indicated that neuron activity regulates the shape of dendritic spine.

In addition, to investigate the influence of exogenous activator (δ_A_) and exogenous inhibitor (δ_H_) on the shape of dendritic spine, we adjusted the value of parameter δ_A_ and δ_H_ in Equation (2), respectively. We varied the values of δ_A_ under the conditions of ε = 0.03, 0.05, 0.7, and 0.9 and the values of δ_H_ under the conditions of ε = 0.01, 0.03, 0.07, and 0.7, and the results are shown in Figures 4B,C (also see Supplementary Figures 3, 4, respectively). According to the results, we found that a stubby-type spine transforms to mushroom-type and a thin-type spine transforms to stubby-type with an increase in δ_A_; additionally, a branched-type spine becomes thin-type with an increase in δ_H_. However, there is no effect of δ_A_ on branched-type spines and no effect of δ_H_ on mushroom-type and stubby-type spines. These results indicated that both δ_A_ and δ_H_ also regulate the spine shape but they are not dominating factors compared to the consumption rate of substrates.

Therefore, dendritic spines sequentially undergo in-turn transformation of mushroom-type, stubby-type, thin-type, and branched-type, with an increase in the consumption rate of substrates. In contrast, exogenous activators affect non-branched dendritic spines, and exogenous inhibitors affect branched dendritic spines. Thus, the consumption rate of substrates (neuron activity) determines the shape of dendritic spines.

### Dendritic Spine Densities Factors Research Based on Reaction-Diffusion Model

Dendritic spines participate in the formation of most excitatory axodendritic synapses, so the density of spines directly influences the density of synapses. In order to research the factors of dendritic spine density, we simulated a dendrite with spines using the reaction-diffusion model and found the relationship between dendritic spine density and key factors. Moreover, we observed the decrease of spine density in the hippocampal CA1 in rats with glioma and proposed a potential reason for this phenomenon by comparing the simulation results and observation results. Further, we used Turing instability to explain the mathematical mechanism behind the above parameters regulating spine density and found that an exogenous inhibitor and activator changes Turing wavelength through which to regulate spine densities.

#### Exogenous Substances Regulate Spine Density

To investigate the factors of dendritic spine density, we next simulated different spine densities which seen across multiple spines through dendrite simulations. In our previous research, we found that the rates of activator and inhibitor secretion from cells have been shown to play an important role in the density of side branching (Guo et al., 2014a). Similarly, it is reasonable for us to assume that exogenous activator and inhibitor are two key factors influencing the density of dendritic spines.

**Figure 5:**
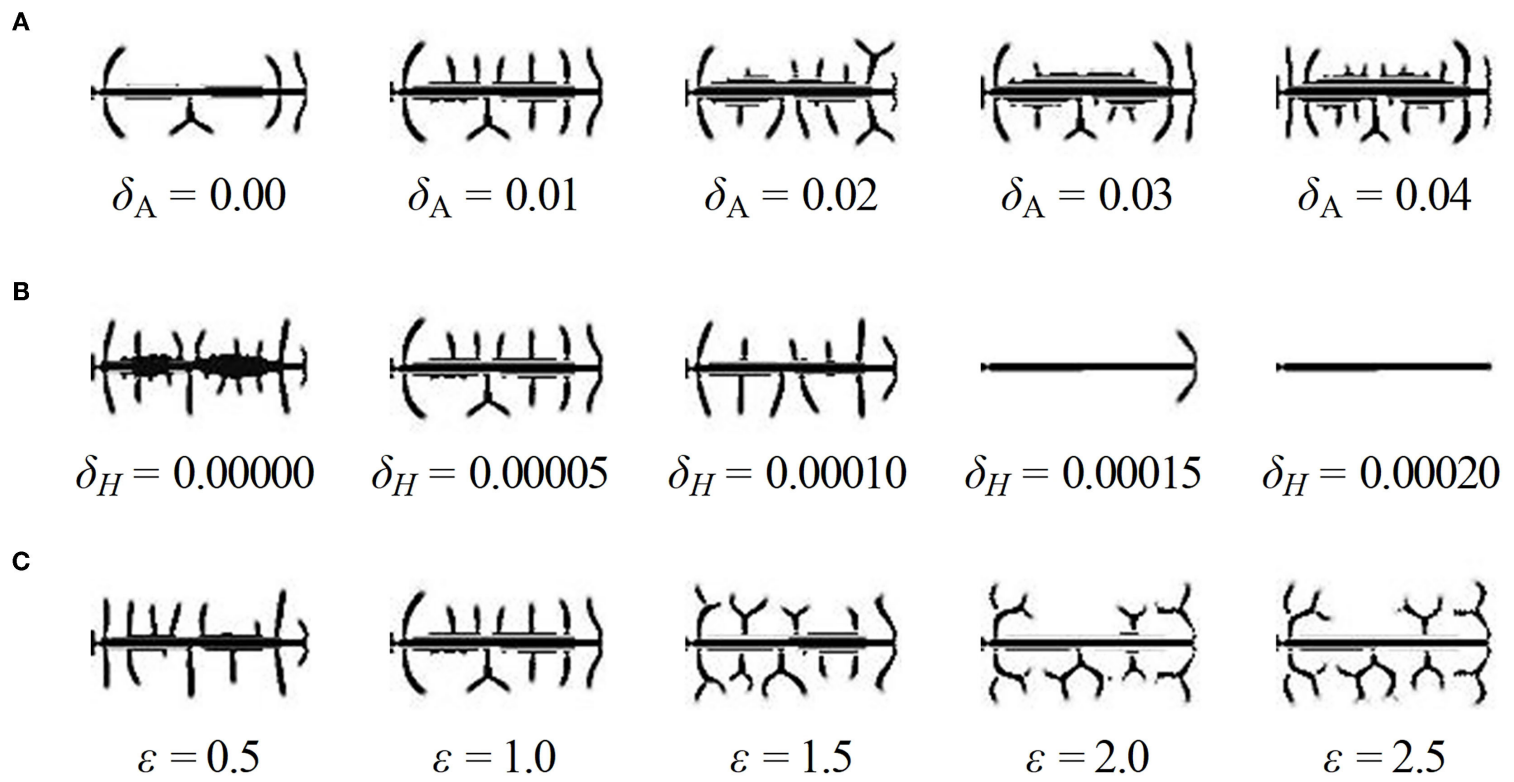
Influence of parameters on the density of dendritic spines. **(A)** The results of dendrite simulation under the conditions of δ_A_ = 0, 0.01, 0.02, 0.03, and 0.04, δ_H_ = 0.00005, ε = 1. With the addition of an exogenous activator, the number of dendritic spines increases dramatically. **(B)** The results of dendrite simulation under the conditions of δ_A_ = 0.01, δ_H_ = 0, 0.00005, 0.0001, 0.00015, and 0.0002, ε = 1. With the addition of an exogenous inhibitor, the number of dendritic spines decreases dramatically. **(C)** The results of dendrite simulation under the conditions of δ_A_ = 0.01, δ_H_ = 0.00005, ε = 0.5, 1, 1.5, 2, and 2.5. With increased neuron activity, the shape varies from non-branched-dominant to branched-dominant. Branched-type spines take up more space; thus, the growth of surrounding spines is inhibited. In addition, the parameter ε has no effect on the density; for example, the densities are the same under the conditions of ε = 2 and ε = 2.5. In **(A–C)**, fixed parameters in Equation (2): *c* = 0.002, μ = 0.16, υ = 0.04, ρ_A_ = 0.02, ρ_H_ = 0.00005, *c*_0_ = 0.05, γ = 0.02, *D*_A_ = 0.02, *D*_H_ = 0.26, *D*_S_ = 0.06, *d* = 0.0035, *e* = 0.1, and *f* = 10.

Firstly, in order to find out the effect of exogenous activator and inhibitor on the spine density, we adjusted the values of the two parameters δ_A_ and δ_H_ based on standard values of δ_A_ = 0.01, δ_H_ = 0.00005, and ε = 1, and we obtained two groups of results (Figures 5A,B). The results showed the density of dendritic spines is positively correlated with δ_A_ and negatively correlated with δ_H_.

Next, we adjusted the values of the parameter ε to find whether the consumption rate of substrates is another factor of density, and the results are shown in Figure 5C (also see Supplementary Figures 1, 2, respectively). We noticed that the spine shape varied from non-branched-dominant to branched-dominant when ε varies from 0.5 to 2.0. Meanwhile, the spine densities have not significant changes when ε varies; for example, the densities are the same under the conditions of ε = 2 and ε = 2.5.

Through dendrite simulations, we found that exogenous activators increase the density of spines, while exogenous inhibitors have the opposite effect. In comparison to exogenous substances, neuron activity has no significant effect on the density.

#### Application in the Hippocampal CA1 of Rats

**Figure 6:**
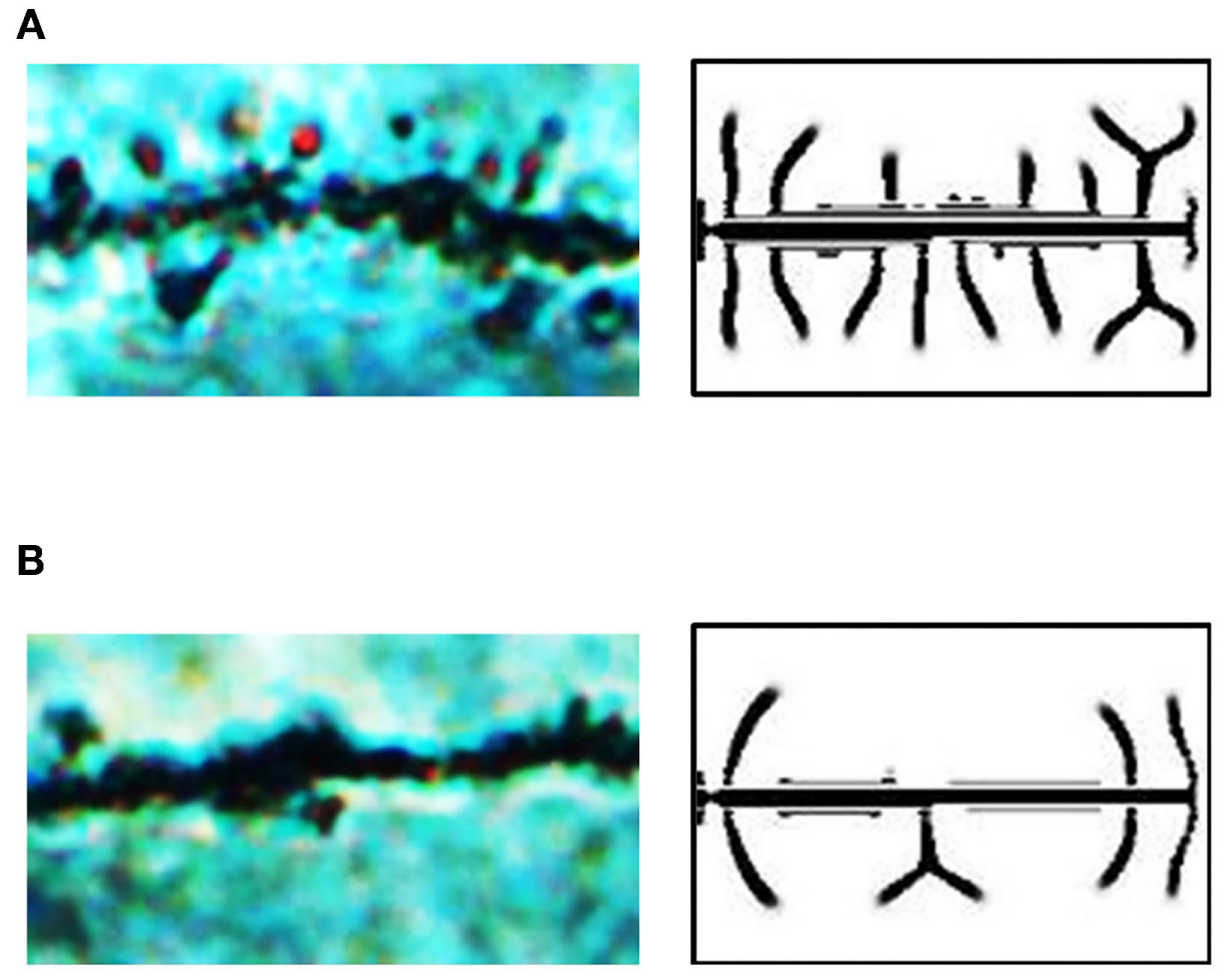
Exogenous inhibitor decreases the spine density in hippocampal CA1 in SD rats with glioma. **(A)** A microscopic image of dendritic spines in the brain of a rat in the sham group. There are dense spines in this image, similar to those in our dendrite simulation results under the condition of δ_A_ = 0.01, δ_H_ = 0, ε = 1. **(B)** A microscopic image of dendritic spines in the brain of a rat in the glioma group. There are sparse spines in this image, similar to those in our dendrite simulation results under the condition of δ_A_ = 0.01, δ_H_ = 0.0001, ε = 1. In **(A,B)**, fixed parameters in Equation (2): *c* = 0.002, μ = 0.16, υ = 0.04, ρ_A_ = 0.02, ρ_H_ = 0.00005, *c*_0_ = 0.05, γ = 0.02, *D*_A_ = 0.02, *D*_H_ = 0.26, *D*_S_ = 0.06, *d* = 0.0035, *e* = 0.1, and *f* = 10.

The hippocampus plays an important role in memory function and cognitive abilities (Muller et al., 1996). Certain diseases, such as glioma, affect the developmental patterns of dendritic spines on hippocampal neurons. It has also been reported that the impairment of neurocognitive function is a common consequence of glioma, in both glioma patients (Wefel et al., 2016; Van Kessel et al., 2017) and glioma animal models (Wang et al., 2010; Hao et al., 2018). Through anatomy and neuron microimaging (see section Anatomy of Hippocampal CA1 in SD Rat for detail), we found that dendritic spines in rats with glioma were less dense (Figures 6A,B, also see Supplementary Videos 5, 6, respectively).

To study the reasons for various densities in the rat hippocampal CA1, we compared the microscopic images of neurons with our simulation results. It seems that the spine patterns in the brains of the rat sham group were similar to those in the simulation results under the condition of δ_A_ = 0.01, δ_H_ = 0, ε = 1, while the spine patterns in the brains of the rat glioma group were similar to those in the simulation results under the condition of δ_A_ = 0.01, δ_H_ = 0.0001, ε = 1 (Figure 5B). Thus, we considered that the addition of exogenous inhibitors is a potential reason for the decrease of dendritic spine density caused by glioma.

#### Turing Instability Underlying Dendritic Spines

Turing pointed out that the diffusion of chemical substances will break the original equilibrium state of substance concentration, which is called Turing instability (Turing, 1952). Branching patterns can only be generated from models with Turing instability. In order to qualitatively analyze the Turing instability, equilibrium position, and periodicity of the model solution, we have proposed a Turing instability analysis method using dispersion relation in previous research (Guo et al., 2014b) and found that the Turing wavelength is the internal factor causing the change of branching pattern of a lung (Xu et al., 2017) (see section Turing Instability Analysis Method for more details).

**Figure 7:**
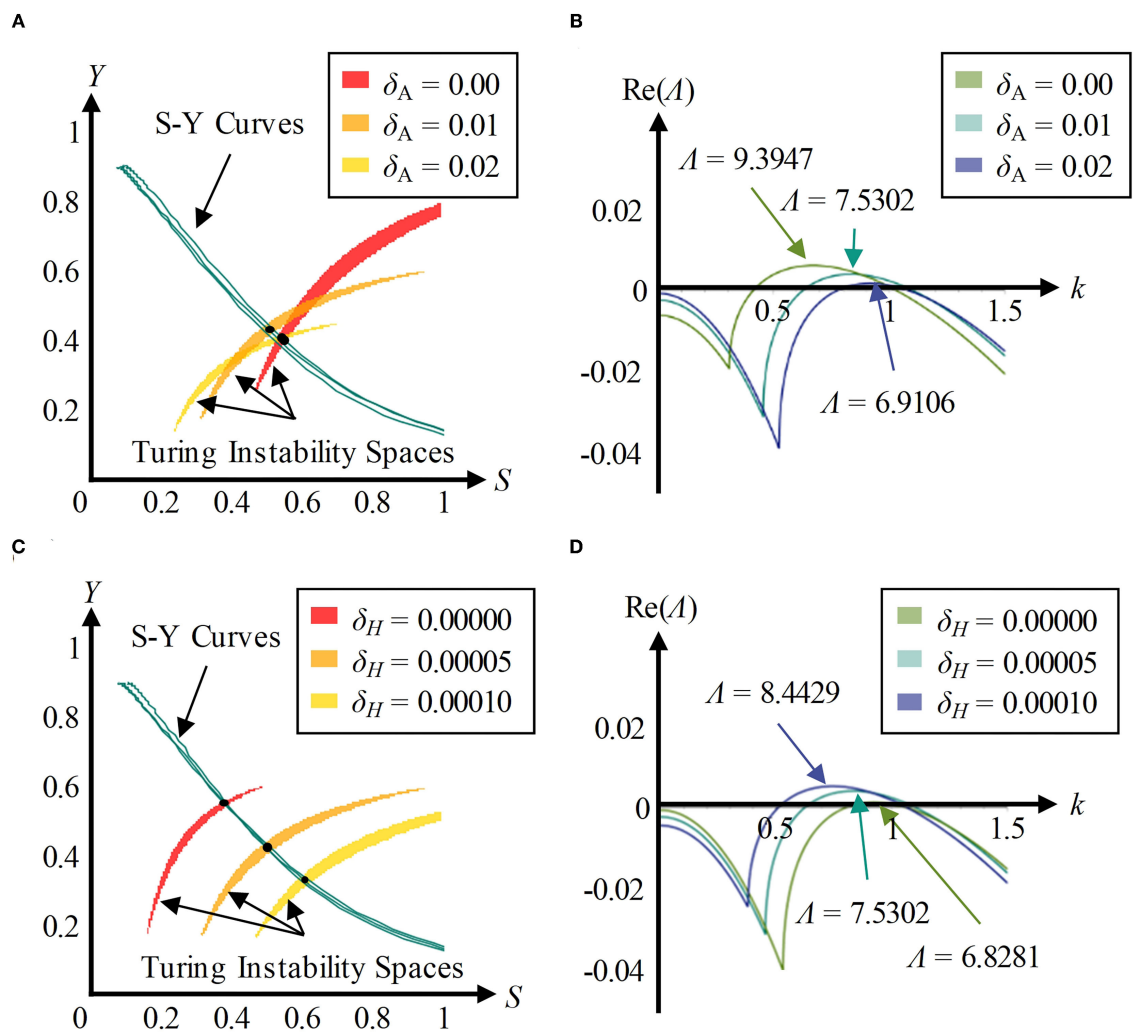
Exogenous substances regulate dendritic spine density through Turing wavelength. **(A)** The S-Y curve and Turing instability space in three conditions of parameter δ_A_ (δ_H_ = 0.00005, ε = 1). The yellow, orange, and red regions represent Turing instability spaces in the conditions of δ_A_ = 0, 0.01, and 0.02, respectively. The cyan lines represent S-Y curves, and the black points represent the intersections. An exogenous activator affects the size and location of the Turing instability space, rather than the S-Y curves (artificial errors in choosing intersections result in non-coincidence). **(B)** The dispersion relationship curves in the conditions of δ_A_ = 0, 0.01, and 0.02 (δ_H_ = 0.00005, ε = 1). Adding an exogenous activator decreases the Turing wavelength. **(C)** The S-Y curve and Turing instability space in three conditions of parameter δ_H_ (δ_A_ = 0.01, ε = 1). The yellow, orange, and red regions represent Turing instability spaces in the conditions of δ_H_ = 0, 0.00005, and 0.0001, respectively. The cyan lines represent S-Y curves, and the black points represent the intersections. An exogenous inhibitor affects the size and location of the Turing instability space, rather than the S-Y curves (artificial errors in choosing intersections result in non-coincidence). **(D)** The dispersion relationship curves in the conditions of δ_H_ = 0, 0.00005, and 0.0001 (δ_A_ = 0.01, ε = 1). Adding an exogenous inhibitor increases the Turing wavelength.

Exogenous substances have an effect on the Turing instability space in which a stable pattern can appear, and have no effect on the *S*-*Y* curve that shows the concentration relationship between substrate and cytoskeleton during development, which can be derived from Equation (3). We adjusted the values of the parameters δ_A_ from 0 to 0.2 and then drew an *S*-*Y* curve and Turing instability space in the *S*-*Y* space (Figure 7A). Three intersection points of Turing instability spaces and the corresponding *S*-*Y* curves were marked with black points. These points were substituted into the Equations (4–6) in order to calculate the Turing wavelengths (Figure 7B). An increase in parameter δ_A_ decreases the Turing wavelength. Similarly, we found the intersection points and calculated Turing wavelength under the conditions of δ_H_ = 0, 0.00005, and 0.0001 (Figures 7C,D). An increase in parameter δ_H_ increases the Turing wavelength.

As the Turing wavelength implies the spatial periodicity of spines, it is negatively correlated to the density of dendritic spines. In conclusion, exogenous activators make the Turing wavelength smaller and cause an increase in density of dendritic spines, while exogenous inhibitors increase the Turing wavelength and cause a decrease in the density of dendritic spines.

## Discussion

In recent years, various chemicals have been reported to be capable of regulating the process of dendritic spine development. Our research may explore their regulation mechanism in a mathematical view. For example, the actin filaments (F-actin) were considered to be key in regulating the shape of dendritic spines (Miermans et al., 2017). We found the cytoskeleton was one key factor to regulate cell morphology. Hence, F-actin might be considered as the cytoskeleton (*Y*) in our model. It has been found that drebrin is an actin-binding protein in the dendritic spine, and its overexpression causes spine elongation (Hayashi and Shirao, 1999; Koganezawa et al., 2017; Hanamura et al., 2018). Bernstein reported that cofilin severs F-actin, contributing to actin dynamics (Bernstein and Bamburg, 2010). In addition, Calabrese suggested that dendritic spine growth correlates with decreased cofilin activity (Calabrese et al., 2014). According to our simulation results, drebrin and cofilin are similar to the functions of the activator (*A*) and the inhibitor (*H*) in our model respectively. Adenosine-triphosphate (ATP) is closely related to F-actin polymerization and depolymerization (Katkar et al., 2018; Merino et al., 2018), which implies that ATP may correspond to the substrate (*S*) in our model. Based on these hypotheses, we described our inferences as follows: (1) the overexpression of drebrin promotes the binding of F-actin and increases the density of dendritic spines, (2) the overexpression of cofilin hinders the binding of F-actin and decreases the density of dendritic spines, (3) the increase in ATP consumption during the process of creating F-actin results in a different F-actin pattern and causes spines to become mushroom-type, stubby-type, thin-type and branched-type, in turn.

The verification experiments of morphogens is helpful to the correction of model parameters and the support of the conclusion in this work. Here, we proposed two ideas to verify the morphogens mentioned above: (1) research on the quantitative relationship between spine density and the addition of a substance that influences the expression of drebrin or cofilin, and (2) research on the quantitative relationship between spine shape distribution and ATP consumption during the process of creating F-actin. Moreover, in order to compare the spatiotemporal parameters between simulations and verification experiments quantitatively, 3D simulation is necessary.

With our method, certain diseases could be systematically investigated at the level of chemical reactions. For example, the anomalous rise of rho GTPase Rac1 activity inhibited cofilin in mice with Fragile X syndrome because of a trinucleotide expansion in the FMR1 gene on the X chromosome (Pyronneau et al., 2017). In our model, the decrease in δ_H_ decreases the concentration of the inhibitor (*H*), which results in dense dendritic spines. In another study, the intrathecal administration of latrunculin A, an actin-depolymerizing agent, in mice resulted in a decrease in F-actin levels and symptoms of Alzheimer's disease. Conversely, the intrathecal administration of jasplakinolide, a molecule that stabilizes F-actin, in mice restored F-actin levels and improved symptoms (Kommaddi et al., 2018). The effects of latrunculin A and jasplakinolide are similar to those of exogenous inhibitors and exogenous activators in this model, respectively. Exogenous activators promote the synthesis of the cytoskeleton, while exogenous inhibitors promote the decomposition of the cytoskeleton.

In conclusion, we were devoted to revealing the mechanism of the development patterns of dendritic spines. The results show that the consumption rate of substrate dominates the shape, while the addition of exogenous activators and exogenous inhibitors dominates the density. Our work provided a potential explanation for the phenomenon that sparser spines in the brains of SD rats with glioma and maybe also explain some diseases reported in the literature, such as Fragile X syndrome and Alzheimer's disease. Our research provides novel and fresh insight into the development patterns of dendritic spines, helping search treatment methods for related diseases.

## Data Availability Statement

The original contributions presented in the study are included in the article/Supplementary Material, further inquiries can be directed to the corresponding authors.

## Ethics Statement

The animal study was reviewed and approved by Animal Research Ethics Committee, School of Medicine, Nankai University.

## Author Contributions

YJ: conceptualization, methodology, software, formal analysis, investigation, writing-original draft, writing-review and editing, and visualization. QZ: conceptualization, methodology, writing-original draft, writing-review and editing, and funding acquisition. HY: methodology, validation, formal analysis, investigation, writing-original draft, writing-review and editing, and visualization. SG: methodology, software, formal analysis, data curation, and writing-review and editing. MS: validation, formal analysis, writing-review and editing, and funding acquisition. ZY: conceptualization, writing-review and editing, and supervision. XZ: conceptualization, writing-review and editing, supervision, project administration, and funding acquisition. All authors contributed to the article and approved the submitted version.

## Conflict of Interest

The authors declare that the research was conducted in the absence of any commercial or financial relationships that could be construed as a potential conflict of interest.
