# Supplementary material for: Reaction–Diffusion Model-Based Research on Formation Mechanism of Neuron Dendritic Spine Patterns
Source: Front Neurorobot. 2021 Jun 14;15:563682. doi: 10.3389/fnbot.2021.563682 (PMC8236519; doi:10.3389/fnbot.2021.563682)
Supplement: Supplementary file 7 [file Data_Sheet_1.DOCX]

Supplementary Material


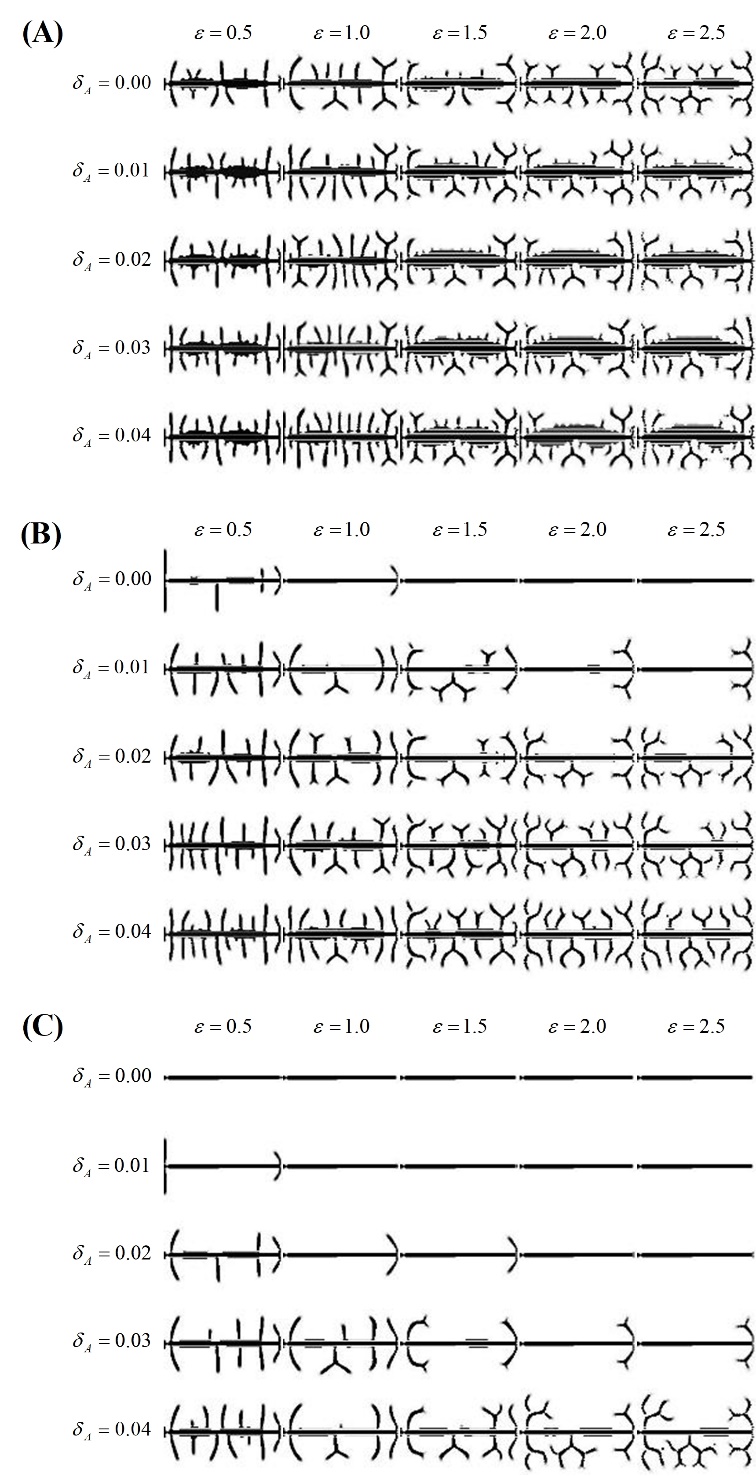


**Supplementary Figure 1.** The simulation results of spines on a dendrite by varying *δ*_A_ and *ε*. **(A)** *δ*_H_ = 0. **(B)** *δ*_H_ = 0.0001. **(C)** *δ*_H_ = 0.0002.


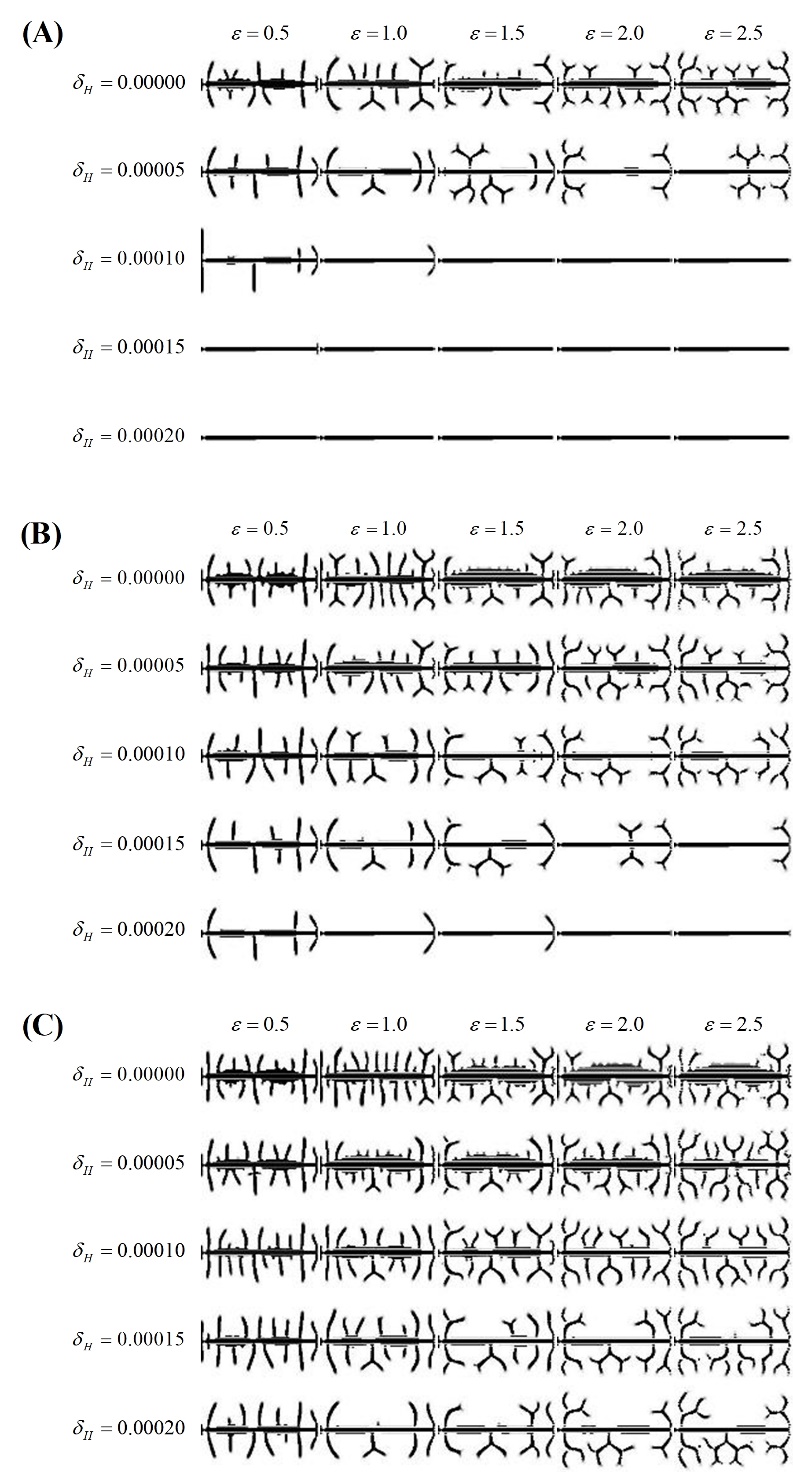


**Supplementary Figure 2.** The simulation results of spines on a dendrite by varying *δ*_H_ and *ε*. **(A)** *δ*_A_ = 0. **(B)** *δ*_A_ = 0.02. **(C)** *δ*_A_ = 0.04.


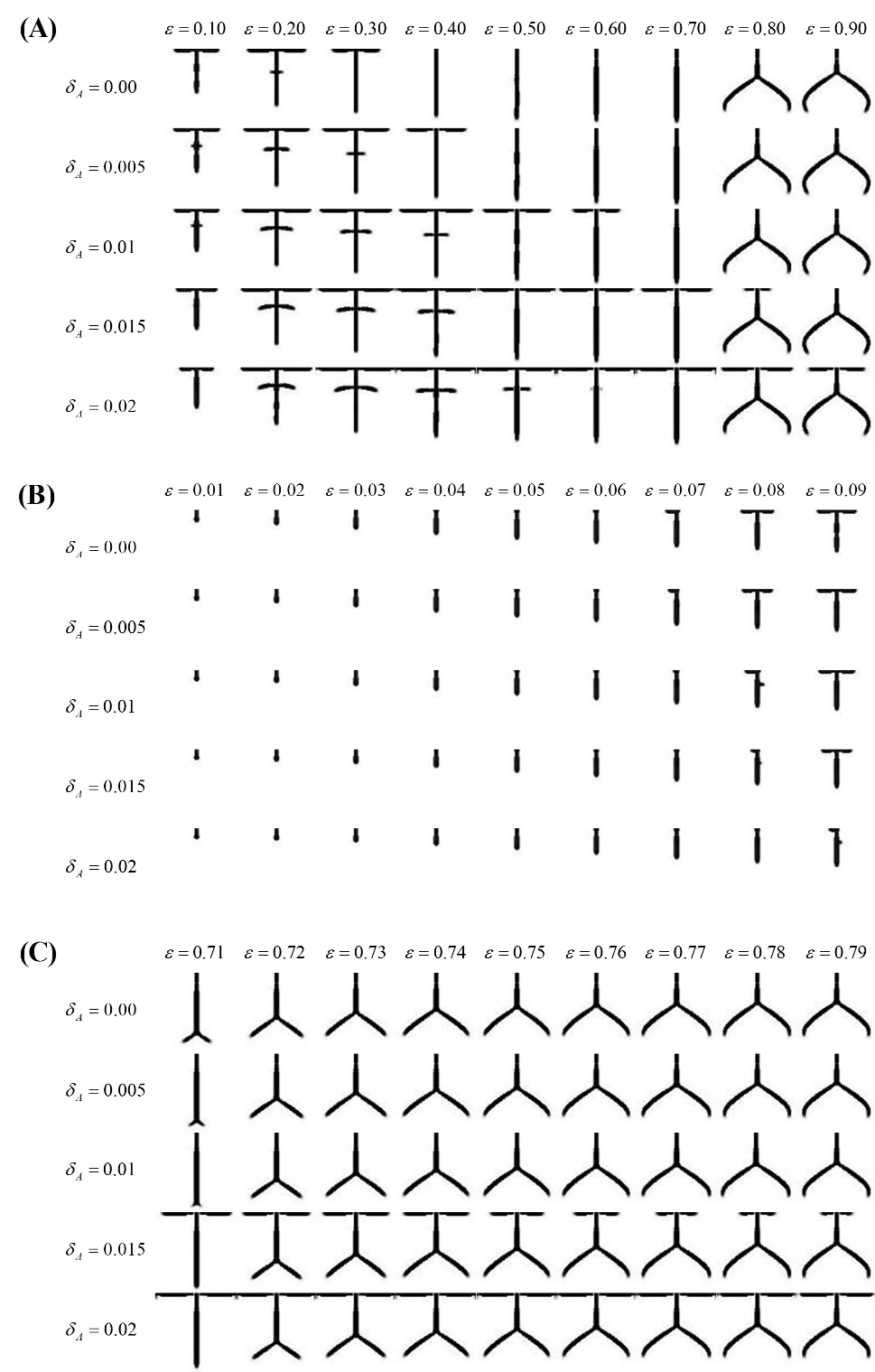


**Supplementary Figure 3.** The simulation results of a spine by varying *δ*_A_ and *ε* (*δ*_H_ = 0.00005). **(A)** 0.1 ≤ *ε ≤* 0.9. **(B)** 0.01 ≤ *ε ≤* 0.09. **(C)** 0.71 ≤ *ε ≤* 0.79.


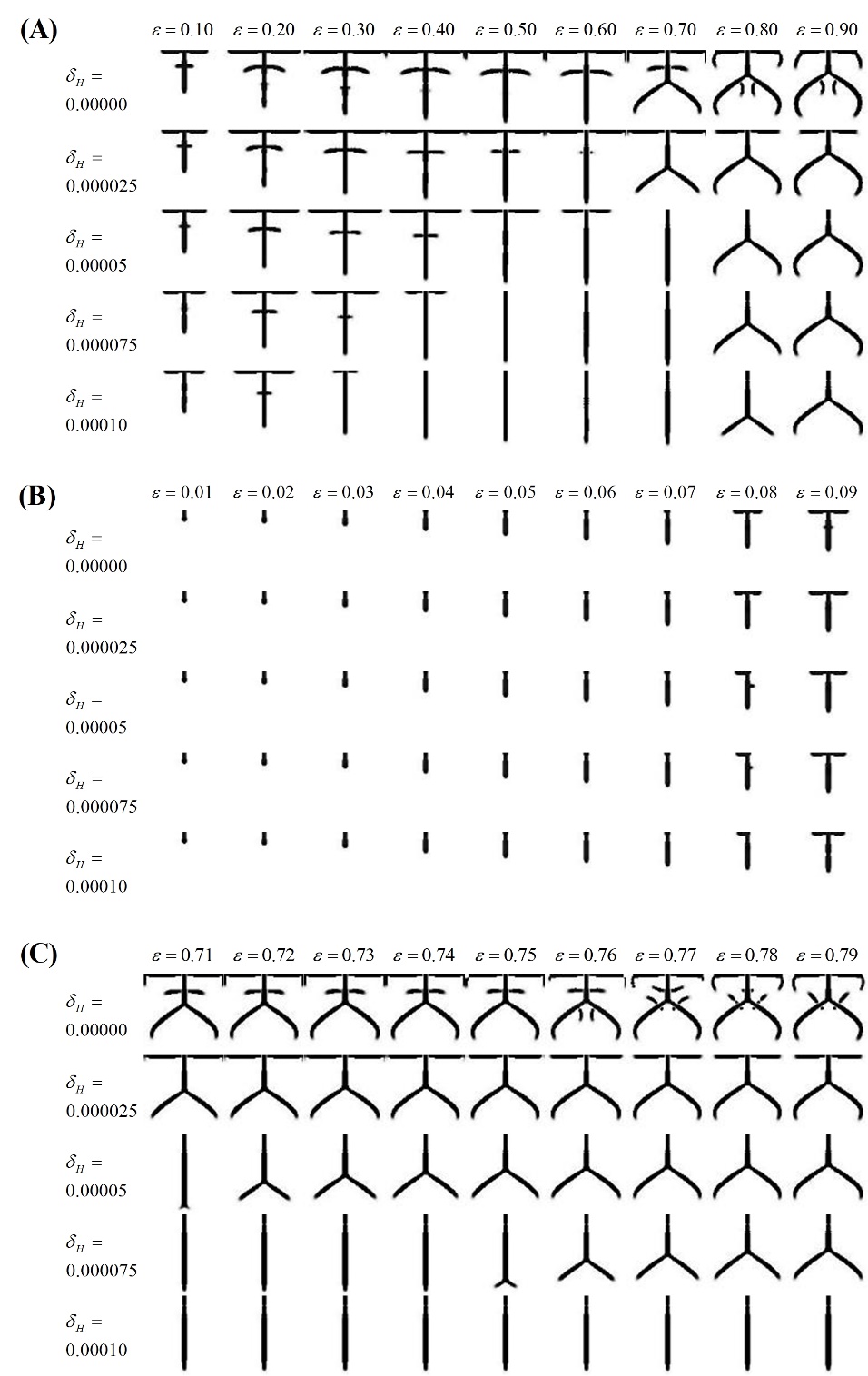


**Supplementary Figure 4.** The simulation results of a spine by varying *δ*_H_ and *ε* (*δ*_A_ = 0.01). **(A)** 0.1 ≤ *ε ≤* 0.9. **(B)** 0.01 ≤ *ε ≤* 0.09. **(C)** 0.71 ≤ *ε ≤* 0.79.

**Supplementary Video 1.** The development process of dense dendritic spine (*δ*_A_ = 0.01, *δ*_H_ = 0.00005, *ε* = 1).

**Supplementary Video 2.** The development process of sparse dendritic spine (*δ*_A_ = 0.01, *δ*_H_ = 0.00015, *ε* = 1).

**Supplementary Video 3.** The development process of mushroom-type dendritic spine (*δ*_A_ = 0.01, *δ*_H_ = 0.00005, *ε* = 0.02).

**Supplementary Video 4.** The development process of stubby-type dendritic spine (*δ*_A_ = 0.01, *δ*_H_ = 0.00005, *ε* = 0.07).

**Supplementary Video 5.** The development process of thin-type dendritic spine (*δ*_A_ = 0.01, *δ*_H_ = 0.00005, *ε* = 0.6).

**Supplementary Video S6.** The development process of branched-type dendritic spine (*δ*_A_ = 0.01, *δ*_H_ = 0.00005, *ε* = 0.8).
